# Fractal Texture Enhancement of Simulated Infrared Images Using a CNN-Based Neural Style Transfer Algorithm with a Histogram Matching Technique

**DOI:** 10.3390/s23010422

**Published:** 2022-12-30

**Authors:** Taeyoung Kim, Hyochoong Bang

**Affiliations:** Korea Advanced Institute of Science and Technology (KAIST), Daejeon 34141, Republic of Korea

**Keywords:** neural style transfer, fractal analysis, synthetic infrared image, MWIR, OKTAL-SE, image quality assessment, image texture enhancement, NIQE, SSIM

## Abstract

Here, we propose a CNN-based infrared image enhancement method to transform pseudo-realistic regions of simulation-based infrared images into real infrared texture. The proposed algorithm consists of the following three steps. First, target infrared features based on a real infrared image are extracted through pretrained VGG-19 networks. Next, by implementing a neural style-transfer algorithm to a simulated infrared image, fractal nature features from the real infrared image are progressively applied to the image. Therefore, the fractal characteristics of the simulated image are improved. Finally, based on the results of fractal analysis, peak signal-to-noise (PSNR), structural similarity index measure (SSIM), and natural image quality evaluator (NIQE) texture evaluations are performed to know how the simulated infrared image is properly transformed as it contains the real infrared fractal features. We verified the proposed methodology using a simulation with three different simulation conditions with a real mid-wave infrared (MWIR) image. As a result, the enhanced simulated infrared images based on the proposed algorithm have better NIQE and SSIM score values in both brightness and fractal characteristics, indicating the closest similarity to the given actual infrared image. The proposed image fractal feature analysis technique can be widely used not only for the simulated infrared images but also for general synthetic images.

## 1. Introduction

Synthetic Infrared (IR) imaging is widely used in military, medical, and industrial fields as a substitute for an actual infrared imaging system [1,2,3,4]. Today, generation of synthetic infrared images is commonly performed by commercial software tools such as MuSES [5], Vega Prime [6], and OKTAL-SE [7]. Moreover, recent texture enhancement techniques such ray-tracing and anti-aliasing in computer graphics have been adopted to generate realistic synthetic infrared images [8,9,10]. Despite the completeness of synthetic infrared images, the fundamental question of how to depict low-level thermal components, such as rocks and leaves, without massive computational loads remains. In general, the background elements are replaced with repetitive texture components instead of direct modeling and individual material allocation. Therefore, we propose a neural style transfer-based texture enhancement algorithm for simulated infrared images with a histogram matching technique. Since this method uses a single real infrared image as a style reference, it can reduce computational load and acquisition cost for a target infrared image. Specifically, the proposed stylization technique combines brightness histogram information with the fractal texture features of a real infrared images.

The main contribution of this paper can be summarized as follows:

- A modified neural style transfer algorithm with a histogram matching technique is proposed for realistic synthetic infrared image generation.
- The image quality assessment procedure with a fractal image along with a false-colored RGB infrared image is suggested.
- The natural image quality index (NIQE) and structural similarity index measure (SSIM) are proposed as evaluation metrics for synthetic infrared images.

The rest of the paper is organized as follows. Section 2 gives a brief description of the related work. Section 3 presents the proposed fractal dimension-based image enhancement for simulated infrared images. Section 4 shows the simulation results according to three different simulation conditions. Finally, we conclude with a summary in the last section.

## 2. Related Works

### 2.1. Simulated Infrared Image

To overcome hardware limitations in infrared imaging, numerical simulations of the thermal distribution of the aircraft skin and exhaust gases have been widely researched using commercial CFD software [11] with sophisticated mathematical computer code for configuring a realistic simulation environment [12]. In recent studies, simulated infrared imaging using a graphics engine such as Unity 3D has also expanded its scope to a VR/AR virtual environment [13,14]. In this way, infrared radiation characteristics in various observation band wavelengths and directions can be easily analyzed in a physics model-based simulation. However, these physics-based simulations have intrinsic limitations in that they cannot describe all the various real-world environments due to the constraints of computation resources.

### 2.2. Neural Style Transfer

Recently, generative model-based approaches such as CycleGAN have been researched for texture enhancement of simulated infrared images [15,16]. However, the generative models have a high risk of distorting the physical context of the original images. In addition, it requires many sample datasets to train the neural networks. On the other hand, a neural style transfer can reduce computational load as it does not require prior knowledge in both domains [17]. Since the seminal work of neural style transfer [18], there have been many recent studies showing that style transfer methods based on convolutional neural network (CNN) are biased toward texture rather than shape [19]. According to the Markov random field theory, traditional texture synthesis is characterized by statistical interactions within local neighborhoods [20]. These statistical interactions are determined according to the oriented linear kernels at multiple spatial scales. The style transfer algorithm matches the content and style representation in the intermediate layers of the images. Therefore, the style of the reference image can be transferred directly to the target image. This process is implemented by optimizing the statistics of the output image according to the statistics of the style image and content image.

### 2.3. Fractal-Based Image Analysis

A fractal is defined as a repetitive pattern of self-similarity found in nature, such as trees, lightning, and rivers, which follows the law of power series. The fractal concept was first studied by mathematician Mandelbrot in 1975 [21]. Fractal structures are used in various fields, such as science, engineering, and computer graphics, as a method of synthesizing nature images or classifying a segment of textures [22]. For example, Pentland [23] modeled the surface of natural structures found in mountains, trees, and clouds as fractals by using computer graphic tools. To distinguish natural images and artificial images, Pentland [23] also defined the fractal dimension that changes according to scale size as a fractal signature used for texture classification. The fractal dimension (FD) is an important characteristic parameter of fractal structures that measures the level of self-similarity of patterns. This quantitative fractal information tells us about how much the geometric structure follows the power law of scale invariance. In practice, the fractal dimensions are used to measure the pavement surface resistance evaluation [24]. Moreover, the value of the fractal dimension can be used as a fractal signature for the classification of image textures [25,26]. Therefore, we use this idea as a key characteristic that distinguishes the simulated infrared image from the actual infrared image.

## 3. The Proposed Algorithm

**Figure 1:**
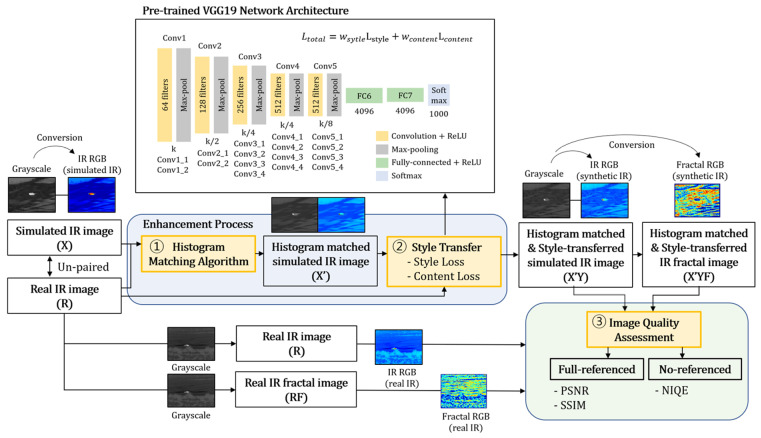
Flow chart of the proposed texture enhancement process of a simulated infrared image.

In this paper, we propose the neural style transfer algorithm using histogram matching, which matches the infrared characteristics of the real infrared image in the perspective of both thermal signature and texture. Therefore, the overall brightness of the real infrared image with fractal characteristics can be transmitted to the simulated infrared image. The generated style-transferred simulated infrared images, as a result of the statistical correlations, were evaluated using both the full-reference image quality metrics and the no-reference image quality metrics. A diagram of the proposed algorithm is given in Figure 1.

The algorithm uses three infrared images to calculate the total loss: the histogram-matched simulated IR image $X^{\prime }$ (input), the style-transferred simulated infrared image Y (output), and the real infrared image R (style). First, the input histogram-matched simulated infrared image ($X^{\prime }$) and the style-transferred infrared image ($X^{\prime }Y$) are passed to the pretrained VGG-19 networks, and the multi-layered features are extracted. Because CNNs are successively located in each block of the VGG-19 networks, it has the advantage of preventing loss of information of the image. The content loss is calculated by using the spatial features of the input image ($X^{\prime }$) and the output image ($X^{\prime }Y$). In addition, style loss is calculated by using the style characteristics of the output image ($X^{\prime }Y$) and the real infrared image (R). Therefore, the total loss can be calculated by the weighted sum of those loss terms.

- Content loss, L($X^{\prime }$*,* $X^{\prime }Y$): Content loss is a measure of the difference in spatial structure between the histogram-matched simulated infrared image ($X^{\prime }$) and the style-transferred simulated infrared image ($X^{\prime }Y$).
- Style loss, L($R,\;X^{\prime }Y$): Style loss is a measure of similarity in the stylistic features between the real infrared image (R) and the style-transferred infrared image ($X^{\prime }Y$).

The infrared image quality assessment process can be divided into three steps. First, both the actual infrared image and the generated output infrared image are converted to false-colored infrared RGB images in order to utilize the enhanced feature information in each scene. Next, the fractal RGB images are generated to check the spurious simulation-like regions from the infrared images. Finally, a quantitative evaluation using the full-reference image quality metrics is conducted based on the peak signal-to-noise (PSNR) and structural similarity index measure (SSIM) of both the generated infrared RGB images and fractal RGB images, as depicted in Figure 1. Moreover, no-reference based image assessment using natural image quality evaluator (NIQE) is performed for the output images to check natural scene statistics. From the results of evaluation in the last phase, it can be decided whether the style-transferred infrared images correctly reflect the infrared features of the given true infrared image. The following subsections describe each of these phases in detail.

### 3.1. Style Matching

Gatys et al. [18] first proposed a CNN-based stylization algorithm for any two images. During the stylization, the correlations between the extracted features defined by the Gram matrix are matched through the optimization framework. The features of the content image are projected to the eigenspace of the features of the style image, and the second-order statistics of each infrared image are matched in the feature space domain. Therefore, the final stylized infrared image is obtained when the extracted features are sufficiently saturated at each feature layer (i.e., conv1_x, conv2_x, conv3_x, and conv4_x).

### 3.2. Content Loss

Content loss is defined as the mean square error (MSE) value of the spatial feature information including the same content of the input image as the output image. The content loss compares the features of the simulated infrared image and the style-transferred infrared image as

(1) $$L_{\mathrm{content}}=E\left({\left[\phi_{l}\left(X^{\prime }\right)-\phi_{l}\left(X^{\prime }Y\right)\right]}^{2}\right),$$

where $X^{\prime }$ is the histogram-matched simulated infrared image, $X^{\prime }Y$ is the style transferred infrared image, and $\phi_{l}$(∙) represents the extracted feature at layer (l).

### 3.3. Gram Matrix

According to Gatys et al. [18], the style of an image can be explained by the correlation between the average of each feature map. It can be obtained by the average of the inner product of the feature vectors; therefore, the Gram matrix is used as a stylistic representation for the image and contains information about which features activate together. The size of the Gram matrix is [C, C] where C is the number of channels. Each entry of the Gram matrix represents the correlation between channels as

(2) $$G_{ij}^{l}\left(\phi_{l}\right)=\frac{1}{H\times W}\sum_{h=1}^{H}\sum_{w=1}^{W}\phi_{l}\left(h,\;w,\;c_{i}\right)\phi_{l}\left(h,w,c_{j}\right),$$

where H is the height of an image, and W is the width of an image.

### 3.4. Style Loss

The role of style loss is to evaluate whether the texture of a style-transferred infrared image is consistent with the texture of the real infrared image. Therefore, the MSE of Gram matrices between two infrared images is defined as the style representation distance of those images. The feature extraction layers can be changed according to the target infrared image as follows:

(3) $$L_{style}=\frac{1}{4C^{2}}\sum_{l=1}^{N_{l}}{\left|G_{ij}^{l}\left(\phi_{l}\left(R\right)\right)-G_{ij}^{l}\left(\phi_{l}\left(X^{\prime }Y\right)\right)\right|}^{2},$$

where j is the index of each feature layer, C is the number of channels, $G_{ij}^{l}\left(\phi_{l}\left(R\right)\right)$ is the Gram matrix of the real infrared image, and $G_{ij}^{l}\left(\phi_{l}\left(X^{\prime }Y\right)\right)$ is the Gram matrix of the style transferred infrared image.

### 3.5. Total Loss

Total loss is defined as a weighted sum of the content loss and the style loss. The image update is performed at every iteration using the *L-BFGS-B* algorithm [27]. The relative weight parameters for $w_{c}=1$ and $w_{s}=10^{6}$ were used in the simulation as follows:

(4) $$L_{total}=w_{c}L_{content}+w_{s}L_{style}.$$

### 3.6. Calculation of Fractal Dimension

Texture is one of main characteristics of infrared images; it defines the particular patterns between the grayscale values of the pixels in a specific region of the image. A texture generally refers to simple image elements that are repeated with small arbitrary changes in color, direction, size, and position throughout the image [28]. Based on the method proposed by Pentland [29], the fractal dimension is evaluated through the regional change of the brightness value according to the change of the displacement vector within the image and used as an index for identifying infrared image traits.

The image intensity difference-based fractal dimension calculation is as follows [23,30]. The intensity of an infrared image of N by M pixels is given by Equation (6), and a displacement vector (w) is defined as in Equation (7). Then,

(5) I=I(x,y),

where

(6) 0≤x≤N−1, 0≤y≤M−1,w=(Δx, Δy) Δx, Δy are integers.

The difference of image intensity at point (x, y) for a displacement vector (w) is defined as $\Delta I_{w}$ and is given by Equation (8):

(7) $$\Delta I_{w}\left(x,y\right)=\Delta I_{w}=I\left(x,y\right)-I\left(x+\Delta x,y+\Delta y\right).\;$$

Second-order statistics of the image equation are defined as in Equation (8). If the logarithm of both sides of Equation (8) is taken, Equation (9) can be acquired. Since the topological dimension of the infrared image is two, the relations between the fractal dimension (D) and the parameter (r) can be summarized as in Equation (11). Finally, the fractal dimension of $\Delta I_{w}$ at point (x, y) for a specific displacement vector (w) is given by Equation (12).

(8) $$E\left(\left|\Delta I_{w}\right|\right)\times {\left|w\right|}^{r-1}=E\left(\left|\Delta I_{ref}\right|\right),$$

where

r=1−H,

(9) $$log\left(\left|\Delta I_{w}\right|\right)+\left(r-1\right)log\left(\left|w\right|\right)=log\left(\left|\Delta I_{ref}\right|\right),$$

(10) $$r=1+\left\{log\left(\left|\Delta I_{ref}\right|\right)-log\left(\left|\Delta I_{w}\right|\right)\right\}/\log\left(\left|w\right|\right),\;$$

(11) D=E+r=2+r, 

(12) $$D\left(w\right)=3+\left\{log\left(\left|\Delta I_{ref}\right|\right)-log\left(\left|\Delta I_{w}\right|\right)\right\}/\log\left(\left|w\right|\right).\;$$

The pseudocode for the generation of the fractal image is summarized in Algorithm 1: Pseudocode for the generation of a fractal image [31].
**Algorithm 1 Generate_fractal_image**1:I ← input infrared image (N×M)
2:w ← dispacement vector (1×p)
3:d ← window size (n×n)
4:$I_{Fractal}$ ← output infrared Fractal image (N×M)
5:**procedure** Generate_fractal_image(I, w, d)6: **for**
$\varepsilon \in \left\{w_{1},\;\cdots w_{p}\right\}$
**do**7:   $\Delta I_{ref}\leftarrow \underset{N\times M}{\sum }\left|I\left(x,y\right)-I\left(x\pm 1,y\pm 1\right)\right|$
8:   $\Delta I_{w}\leftarrow \underset{N\times M}{\sum }\left|I\left(x,y\right)-I\left(x\pm \varepsilon ,y\pm \varepsilon \right)\right|$
9:   $\Delta I_{ref}\leftarrow \mathrm{conv}2d\left(\Delta I_{ref},\;d\right)$
10:   $\Delta I_{w}\leftarrow \mathrm{conv}2d\left(\Delta I_{w},\;d\right)$
11:   $D_{w}\leftarrow 3+\left\{log\left(\Delta I_{ref}\right)-log\left(\Delta I_{w}\right)\right\}/\log\left(\varepsilon \right)$
12: **end for**13: **return**
$I_{Fractal}$ ← average($D_{w})$
14:**end procedure**

## 4. Simulation Results

### 4.1. Dataset Preparation

For the simulation, OKTAL SE-Workbench [7] is used as a tool for modeling the synthetic environment and generating simulated mid-wave infrared (MWIR) images. The image generation conditions for the simulations are shown in Table 1 below.

**Figure 2:**
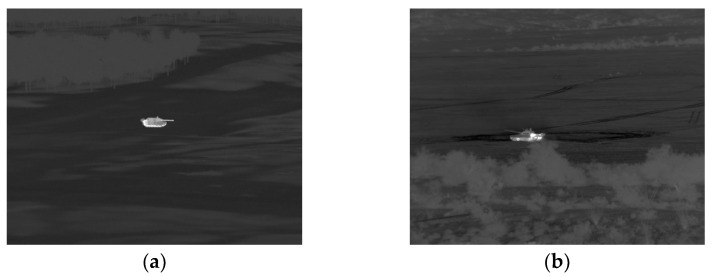
MWIR image examples: (**a**) Simulated infrared image generated with OKTAL-SE [7]; (**b**) Real infrared image from SENSIAC ATR database [32].

The resolution of the generated infrared images is 640 × 480, and the specification of the infrared camera is set to have a fixed f-value with 3.4° × 2.6° FOVs. The distance to the target was assumed to be 1 km and 1.5 km in five different directions: 0°, 70°, 140°, 210°, and 280°. In addition, both seasonal effects and weather effects were considered for making various background texture conditions. Therefore, an example of the simulated infrared image is shown in Figure 2 below.

### 4.2. Implementation Details

In this simulation, a fractal image was generated in the MATLAB R2022a environment [31]. The window size was set to 13, and the size of the displacement vector varied from 3 to 11. Both a real infrared image and a simulated infrared image were randomly selected from the SENSIAC dataset [32] and the aforementioned generated infrared image dataset from OKTAL-SE [7].

### 4.3. Results of the Simple Style Transfer

**Figure 3:**
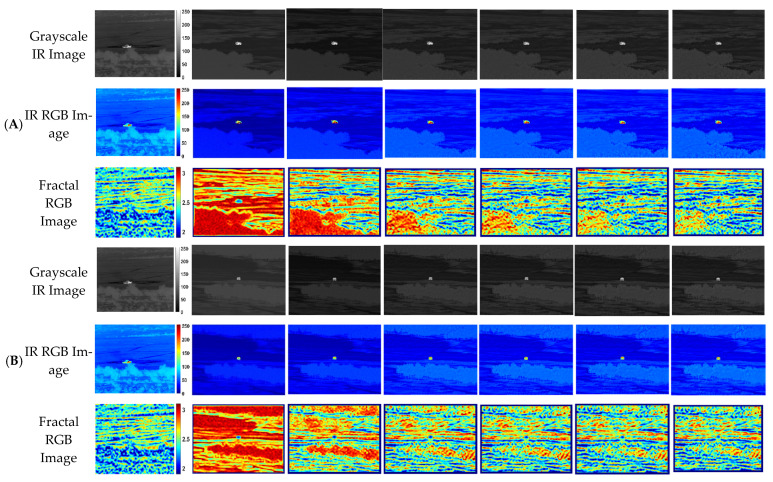

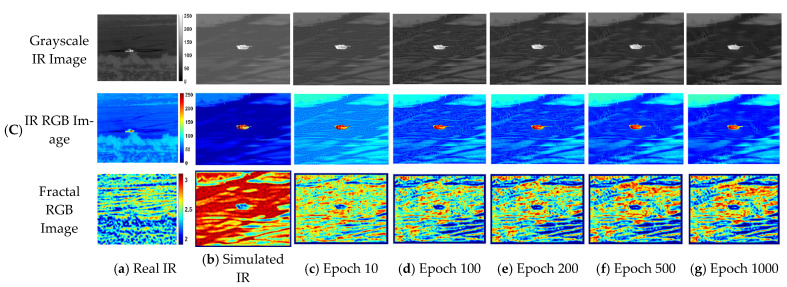
Evolving image features of the grayscale infrared image, false-color infrared image, and fractal image in each of the three simulation conditions: (**A**) Cloudy summer weather at 20 h; (**B**) Fine summer weather at 20 h; (**C**) Cloudy summer weather at 10 h. Each column shows seven different infrared image types: (**a**) Real IR image as a style reference; (**b**) Simulated IR image as a content reference; (**c**) Style-transferred simulated IR image at epoch 1; (**d**) Style-transferred simulated IR image at epoch 100; (**e**) Style-transferred simulated IR image at epoch 200; (**f**) Style-transferred simulated IR image at epoch 500; (**g**) Style-transferred simulated IR image at epoch 1000.

**Figure 4:**
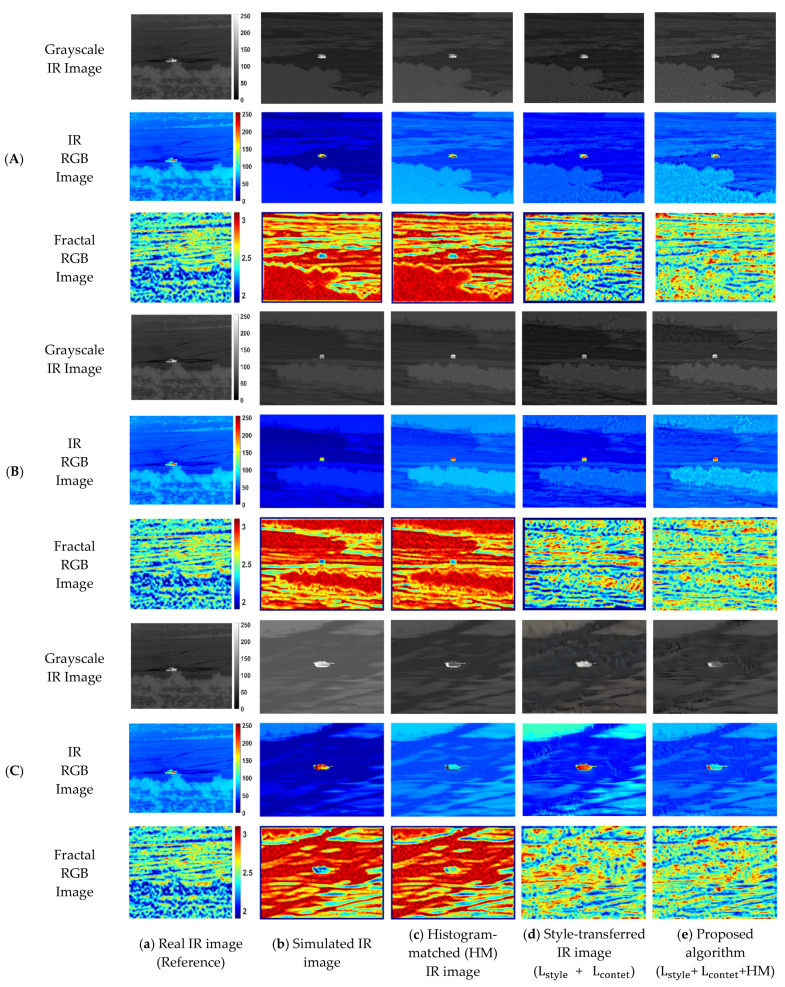
Results of grayscale infrared image, false-colored infrared RGB image, and fractal RGB image in each of the three simulation conditions: (**A**) Cloudy summer weather at 20 h; (**B**) Fine summer weather at 20 h; (**C**) Cloudy summer weather at 10 h with four different image generation techniques: (**a**) Real infrared image as a style reference; (**b**) Simulated IR images; (**c**) Histogram-matched (HM) IR images; (**d**) Neural style transferred IR images; (**e**) Proposed histogram-matched neural style transferred IR images.

Figure 3 shows the application result of the simple style transfer at each epoch. In Figure 4, the false-colored infrared RGB images are also displayed with fractal infrared images. The real IR image of column (a), denoted as a reference, shows a wide fractal distribution due to various background components and thermal interactions with the environment. On the other hand, the simulated infrared image of column (b) shows uniform and high fractal dimension values due to the repetitive random noise patterns on the texture of trees or grassland.

Theoretically, matching the Gram matrix of an image is known as the process of minimizing the mean discrepancy (MMD) using second-order polynomial kernels [33,34]. Therefore, stylization through the Gram matrix is a process in which the second-order polynomial kernels of each image become similar. In other words, the fractal image is consistently related with the Gram matrix minimization process, indicating that the fractal image is more suitable to be used as a quantitative evaluation index of the texture-enhanced (or style-transferred) infrared image rather than the infrared image itself. The qualitative evaluation results in Figure 4 confirm that the background texture of the given simulated infrared images is improved while keeping the physical contents of the original simulated infrared image. Even though they show minor changes in brightness of grayscale infrared images, the fractal images show a clear difference in their distribution and statistics. Therefore, as the epoch progresses, the fractal property from the real infrared image is gradually reflected to the stylized infrared images.

### 4.4. Results of the Proposed Histogram-Matched Style Transfer

Figure 4 show the results of the proposed algorithm compared with other techniques at epoch 1000. Both the simulated infrared images and the histogram-matched simulated infrared images are represented in column (b) and column (c). However, the results of histogram matching in column (c) show that the fractal dimensions are still unchanged from column (b), although the brightness features are similar to the reference infrared image. The result of the simple style transfer in column (d) show that the fractal texture has significantly improved compared to the previous case. Lastly, column (e) shows the result of the proposed algorithm applying histogram matching before the stylization. The advantage of using the histogram matching technique is that the target thermal signature features can be easily transferred. Therefore, by conducting histogram matching, the location of the tank’s heat source (e.g., engine) can be emphasized.

**Figure 5:**
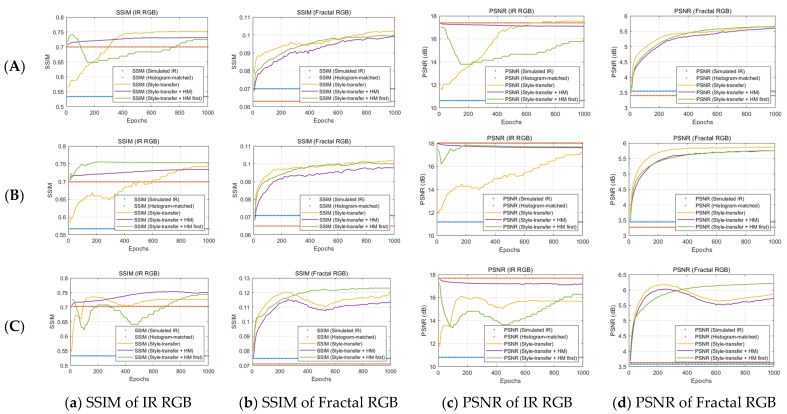
Results of SSIM and PSNR of false-colored IR RGB image and fractal RGB image in each of the three simulation conditions: (**A**) Cloudy summer weather at 20 h; (**B**) Fine summer weather at 20 h; (**C**) Cloudy summer weather at 10 h with the four different image generation techniques mentioned in Figure 4.

For the quantitative evaluation of the results from Figure 5, both SSIM and PSNR changes in epochs were examined. The formulas for SSIM and PSNR follow Equation (13) and Equation (14), respectively.

(13) $$\mathrm{SSIM}\left(x,y\right)=\frac{\left(2\mu_{x}\mu_{y}+c_{1}\right)\left(2\sigma_{xy}+c_{2}\right)}{\left(\mu_{x}^{2}+\mu_{y}^{2}+c_{1}\right)\left(\sigma_{x}^{2}+\sigma_{y}^{2}+c_{2}\right)},\;$$

where $\mu_{x},\;\mu_{y}\;$are local means $\sigma_{x}^{2},\;\sigma_{y}^{2}$ are variance of image X and image Y, and $\sigma_{xy}\;$is the covariance of image X and image Y.

(14) $$\mathrm{PSNR}\;\left(dB\right)=20{log}_{10}\left(\frac{I_{peak}}{\sqrt{MSE}}\right),$$

where

(15) $$\mathrm{MSE}=\frac{1}{H\times W}\sum_{i=0}^{W-1}\sum_{j=0}^{H-1}I\left(i,j\right)-I_{ref}{\left(i,j\right)}^{2},\;I_{peak}=255.$$

As seen in Figure 5, both the SSIM and PSNR on the infrared RGB image and the fractal image are significantly improved compared to the simulated IR image for all cases. In particular, the texture similarity of the style-transferred infrared image can be judged based on the saturated point in the PSNR and SSIM. In addition, SSIM and PSNR features of the fractal images show a smoother and more gradual increase compared to the infrared RGB images, indicating that it can be a stable indicator for checking stylization fidelity. Moreover, from the results of case (B) and case (C) in Figure 5, applying the histogram matching before the style transfer showed better performance in both the PSNR and the SSIM of the fractal RGB images for all cases than the results of applying the histogram matching after the style transfer.

**Figure 6:**
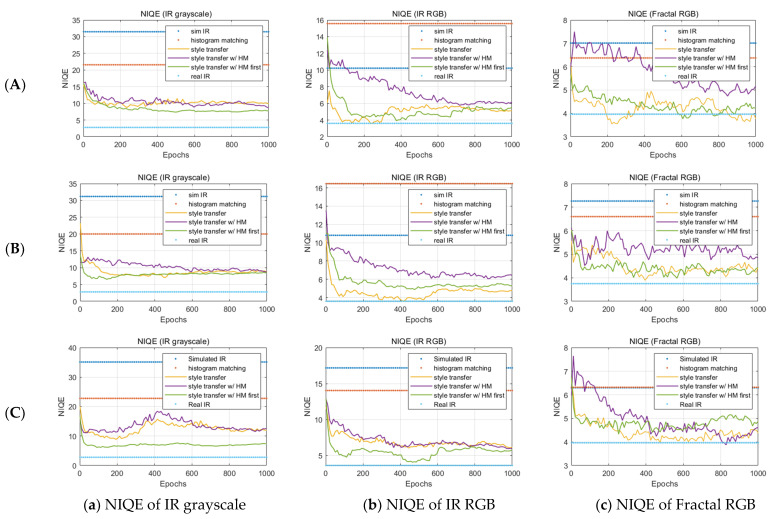
Results of NIQE of grayscale IR image, false-colored IR RGB image, and fractal RGB image in each of the three simulation conditions: (**A**) Cloudy summer weather at 20 h; (**B**) Fine summer weather at 20 h; (**C**) Cloudy summer weather at 10 h with the four different image generation techniques mentioned in Figure 4.

Finally, we plot the evolutionary results of NIQE of the grayscale IR image, IR RGB image, and fractal RGB image to determine the naturalness of the enhanced simulated infrared image in Figure 6. The NIQE scores were measured by the distance between the natural scene statistics (NSS) of resulting infrared images with the real infrared images from the SENSIAC ATR database [32], which was used for training the model. The NSS features are modeled with a multivariate Gaussian distribution (MVG) [35].

(16) $$D\left(\nu_{1},\nu_{2},\Sigma_{1},\Sigma_{2}\right)=\sqrt{{\left(\nu_{1}-\nu_{2}\right)}^{T}{\left(\frac{\Sigma_{1}+\Sigma_{2}}{2}\right)}^{-1}\left(\nu_{1}-\nu_{2}\right)},$$

where $\nu_{1},\nu_{2}\;$and $\Sigma_{1},\Sigma_{2}$ are the mean vectors and covariance matrices of the natural MVG model and the distorted image’s MVG model.

In Figure 6, the resulting infrared image applying the histogram matching before the style transfer showed the lowest NIQE scores during 1000 epochs. This means that the natural statistical characteristics of the resulting infrared images are most similar to the natural image statistical characteristics of the actual infrared image of all the techniques. In addition, the enhancement in NIQE was more evident in the grayscale IR image than in the false-colored IR RGB image. We think that this may be because NIQE strongly measures the naturalness of the undistorted infrared image. The fractal image of column (c) in Figure 6 also shows that the results from the proposed algorithm are most similar to the results of the real infrared image.

## 5. Conclusions

In a rendering pipeline of infrared imaging, natural elements that cause a high computational load, such as tree leaves, pebbles, and bushes are commonly replaced with repetitive textures with random noise. These pseudo-realistic texture elements show high and uniform fractal dimensions unlike the natural background on a real infrared image; therefore, we proposed a CNN-based style transfer algorithm, which matches both fractal and brightness characteristics of the real infrared image to a simulated infrared image through a histogram matching technique.

There are three major contributions of the proposed simulated infrared image enhancement technique. First, to the best of our knowledge, this is a seminal work for evaluating the compatibility of style-transferred simulated infrared images using fractal analysis. Second, we propose using histogram matching, which matches the brightness characteristics in both the style-transferred infrared image and the real infrared image. Therefore, the overall brightness histogram of the real infrared image can be successfully reflected on the style-transferred infrared image without losing physical context and fractal features. Third, the uniform and high-level fractal dimension values of background textures in the simulated infrared image can be regarded as a ‘simulated infrared signature’, which can be used to distinguish natural infrared scenery from the simulated infrared images generated in the virtual-reality environment.

In summary, the proposed algorithm can enhance the simulation-like background texture of simulated infrared images. Specifically, the low-level infrared characteristic drastically improved during stylization. Therefore, both SSIM and NIQE, which are known to be similar to human cognitive appraisal, were greatly improved compared to the results of the naïve simulated infrared images in both infrared and fractal texture characteristics. By utilizing this proposed background texture enhancement method, a limited number of real infrared images can be easily augmented based on an abundance of simulated infrared images.

## Figures and Tables

**Table 1:** Simulated infrared image generation conditions.

Parameter	Values
FOV (°)	3.4 × 2.6 (constant f)
Resolution	640 × 480
Azimuth (°)	0, 70, 140, 210, 280
Range (m)	1000, 1500
Weather	Fine Summer, Cloudy Summer, Fine Fall, Rain Fall
Time (h)	10, 15, 20

## Data Availability

No new data were created or analyzed in this study. Data sharing is not applicable to this article.

## References

[1] Zhang R., Mu C., Yang Y., Xu L. (2018). Research on simulated infrared image utility evaluation using deep representation. J. Electron. Imag..

[2] Sa I., Lim J.Y., Ahn H.S., MacDonald B. (2022). DeepNIR: Datasets for Generating Synthetic NIR Images and Improved Fruit Detection System Using Deep Learning Techniques. Sensors.

[3] Alvey B., Anderson D.T., Buck A., Deardorff M., Scott G., Keller J.M. Simulated photorealistic deep learning framework and workflows to accelerate computer vision and unmanned aerial vehicle research. Proceedings of the IEEE/CVF International Conference on Computer Vision.

[4] Yu G., Zhang G. Real-time simulation of airborne FLIR sensor. Proceedings of the 2016 IEEE Chinese Guidance, Navigation and Control Conference (CGNCC).

[5] MuSES EO/IR Signature Simulation Software. http://www.thermoanalytics.com/products/muses.

[6] Vega Prime. http://www.presagis.com/products_services/products/modeling-simulation/visualization/vega_prime.

[7] Oktal-SE. http://www.oktal-se.fr/.

[8] Richter S.R., Al Haija H.A., Koltun V. (2022). Enhancing Photorealism Enhancement. IEEE Trans. Pattern Anal. Mach. Intell..

[9] Auer S., Hinz S., Bamler R. (2010). Ray-Tracing Simulation Techniques for Understanding High-Resolution SAR Images. IEEE Trans. Geosci. Remote Sens..

[10] Valoroso A.A., White B.C., Ballard J.R., Hunter R.H., Patel R.R. (2020). Massively parallel synthetic sensor-based infrared image generation for object detection. Detection and Sensing of Mines, Explosive Objects, and Obscured Targets XXV.

[11] Willers M.S., Willers C.J. (2012). Key considerations in infrared simulations of the missile-aircraft engagement. Technologies for Optical Countermeasures IX.

[12] MODTRAN® (MODerate Resolution Atmospheric TRANsmission). http://modtran.spectral.com/.

[13] Lahoud F., Susstrunk S. Ar in VR: Simulating Infrared Augmented Vision. Proceedings of the 25th IEEE International Conference on Image Processing (ICIP).

[14] Tran N.C., Wang J.H., Vu T.H., Tai T.C., Wang J.C. (2022). Anti-aliasing convolution neural network of finger vein recognition for virtual reality (VR) human–robot equipment of metaverse. J. Supercomput..

[15] Yun K., Yu K., Osborne J., Eldin S., Nguyen L., Huyen A., Lu T. (2019). Improved visible to IR image transformation using synthetic data augmentation with cycle-consistent adversarial networks. Pattern Recognition and Tracking XXX.

[16] Zhang R., Mu C., Xu M., Xu L., Shi Q., Wang J. (2019). Synthetic IR Image Refinement Using Adversarial Learning With Bidirectional Mappings. IEEE Access.

[17] Gatys L.A., Ecker A.S., Bethge M. Image style transfer using convolutional neural networks. Proceedings of the IEEE Conference on Computer Vision and Pattern Recognition.

[18] Gatys L., Ecker A.S., Bethge M. (2015). Texture synthesis using convolutional neural networks. Adv. Neural Inf. Process. Syst..

[19] Geirhos R., Rubisch P., Michaelis C., Bethge M., Wichmann F.A., Brendel W. (2018). ImageNet-Trained CNNs Are Biased towards Texture; Increasing Shape Bias Improves Accuracy and Robustness. arXiv.

[20] Bela J. (1975). Experiments in the visual perception of texture. Sci. Am..

[21] Mandelbrot B.B. (1975). Stochastic models for the Earth’s relief, the shape and the fractal dimension of the coastlines, and the number-area rule for islands. Proc. Natl. Acad. Sci. USA.

[22] Barnsley M.F., Devaney R.L., Mandelbrot B.B., Peitgen H.O., Saupe D., Voss R.F., Fisher Y., McGuire M. (1988). The Science of Fractal Images.

[23] Pentland A.P. (1984). Fractal-Based Description of Natural Scenes. IEEE Trans. Pattern Anal. Mach. Intell..

[24] Liu C., Zhan Y., Deng Q., Qiu Y., Zhang A. (2021). An improved differential box counting method to measure fractal dimensions for pavement surface skid resistance evaluation. Measurement.

[25] Nirupam S., Chaudhuri B.B. (1992). An efficient approach to estimate fractal dimension of textural images. Pattern Recognit..

[26] Chinmaya P., Seal A., Mahato N.K. (2020). Image texture surface analysis using an improved differential box counting based fractal dimension. Powder Technol..

[27] Zhu C., Byrd R.H., Lu P., Nocedal J. (1997). Algorithm 778: L-BFGS-B: Fortran subroutines for large-scale bound-constrained optimization. ACM Trans. Math. Softw..

[28] Zhou W., Bovik A.C., Sheikh H.R., Simoncelli E.P. (2004). Image quality assessment: From error visibility to structural similarity. IEEE Trans. Image Process..

[29] Pentland A.P. (1986). Shading into texture. Artif. Intell..

[30] Dennis T.J., Dessipris N.G. (1989). Fractal modelling in image texture analysis. IEE Proc. F-Radar Signal Process..

[31] Noah M. (2022). Create, Measure, Characterize, Visualize 1D, 2D, 3D Fractals. MATLAB Central File Exchange. https://www.mathworks.com/matlabcentral/fileexchange/71774-create-measure-characterize-visualize-1d-2d-3d-fractals.

[32] (2008). Military Sensing Information Analysis Center (SENSIAC). https://www.sensiac.org/.

[33] Gonzalez R.C., Woods R.E. (2008). Digital Image Processing.

[34] Li Y., Wang N., Liu J., Hou X. (2017). Demystifying Neural Style Transfer. arXiv.

[35] Anish M., Soundararajan R., Bovik A.C. (2012). Making a “completely blind” image quality analyzer. IEEE Signal Process. Lett..

